# Root Nodule Rhizobia From Undomesticated Shrubs of the Dry Woodlands of Southern Africa Can Nodulate Angolan Teak *Pterocarpus angolensis*, an Important Source of Timber

**DOI:** 10.3389/fmicb.2021.611704

**Published:** 2021-01-28

**Authors:** Wiebke Bünger, Abhijit Sarkar, Jann Lasse Grönemeyer, Janina Zielinski, Rasmus Revermann, Thomas Hurek, Barbara Reinhold-Hurek

**Affiliations:** ^1^Department of Microbe-Plant Interactions, CBIB (Center for Biomolecular Interactions Bremen), Faculty Biology and Chemistry, University of Bremen, Bremen, Germany; ^2^Department of Biodiversity, Ecology and Evolution of Plants, Institute of Plant Science and Microbiology, University of Hamburg, Hamburg, Germany; ^3^Faculty of Natural Resources and Spatial Sciences, Namibia University of Science and Technology, Windhoek, Namibia

**Keywords:** *Pterocarpus angolensis*, *Wiborgia monoptera*, *Indigofera rautanenii*, *Desmodium barbatum*, *Bradyrhizobium*, root nodules

## Abstract

*Pterocarpus angolensis*, a leguminous tree native to the dry woodlands of Southern Africa, provides valuable timber, but is threatened by land conversion and overharvesting while showing limited natural regeneration. Nitrogen-fixing root nodule symbionts that could improve establishment of young seedlings have not yet been described. Therefore, we investigated the ability of *P. angolensis* to form nodules with a diverse range of rhizobia. In drought-prone areas under climate change with higher temperatures, inoculants that are heat-tolerant and adapted to these conditions are likely to be of advantage. Sources of bacterial isolates were roots of *P. angolensis* from nurseries in the Kavango region, other shrubs from this area growing near *Pterocarpus* such as *Indigofera rautanenii*, *Desmodium barbatum*, *Chamaecrista* sp., or shrubs from drought-prone areas in Namaqualand (*Wiborgia monoptera*, *Leobordea digitata*) or Kalahari (*Indigofera alternans*). Only slight protrusions were observed on *P. angolensis* roots, from which a non-nodulating *Microbacterium* sp. was isolated. Rhizobia that were isolated from nodules of other shrubs were affiliated to *Bradyrhizobium ripae* WR4^T^, *Bradyrhizobium* spp. (WR23/WR74/WR93/WR96), or *Ensifer/Mesorhizobium* (WR41/WR52). As many plant growth-promoting rhizobacteria (PGPR), nodule isolates produced siderophores and solubilized phosphate. Among them, only the *Bradyrhizobium* strains nodulated *P. angolensis* under controlled conditions in the laboratory. Isolates were further characterized by multilocus sequence analysis and were found to be distant from known *Bradyrhizobium* species. Among additional reference species tested for nodulation on *P. angolensis*, *Bradyrhizobium vignae* 7-2^T^ and *Bradyrhizobium namibiense* 5-10^T^ from the Kavango region of Namibia as well as *Bradyrhizobium elkanii* LMG6234^T^ and *Bradyrhizobium yuanmingense* LMG21728^T^ induced nitrogen-fixing nodules, while *Bradyrhizobium diazoefficiens* USDA110^T^ and *Bradyrhizobium tropiciagri* SEMIA6148^T^ did not. This suggests a broad microsymbiont range from *Bradyrhizobium japonicum* and *B. elkanii* lineages. Phylogenetic analysis of *nodC* genes indicated that nodulating bradyrhizobia did not belong to a specific symbiovar. Also, for *I. rautanenii* and *Wiborgia*, nodule isolates *B. ripae* WR4^T^ or *Mesorhizobium* sp. WR52, respectively, were authenticated. Characterization of symbionts inducing effective root nodules in *P. angolensis* and other shrubs from Subsahara Africa (SSA) give insights in their symbiotic partners for the first time and might help in future to develop bioinoculants for young seedlings in nurseries, and for reforestation efforts in Southern Africa.

## Introduction

Nitrogen is unique among the other essential plant nutritional elements, as N_2_ from the atmosphere can be fixed by biological nitrogen fixation (BNF), exclusively carried out by prokaryotes that possess the enzyme nitrogenase. The rhizobia-legume symbiosis is a complex and specialized interaction of ecological importance, where nodule symbionts provide nitrogen derived from BNF to the host plant. Making use of BNF by root nodule symbioses can help to alleviate nitrogen deficiencies and increase soil fertility (Pule-Meulenberg et al., 2010), which may be of special importance in low-fertility soils. The key mineral nutrients N and P are in very low concentrations, for example, in sandy soils of Northern Namibia (Gröngröft et al., 2013). In-depth knowledge on rhizobial symbionts in Subsahara Africa is still limited (Grönemeyer and Reinhold-Hurek, 2018), although Africa is a center of origin of many legumes (e.g., cowpea and Bambara groundnut), and offers a rich diversity of wild legume species (Sprent et al., 2010; Pule-Meulenberg, 2014; Lemaire et al., 2015a). Thus, the diversity of *Bradyrhizobium* species in Subsahara Africa may be as yet underestimated and represents a hidden rich resource for inoculant development in future (Grönemeyer and Reinhold-Hurek, 2018).

Several socio-economically important indigenous tree species of Southern Africa are threatened by land conversion and overharvesting, while showing limited natural regeneration (De Cauwer et al., 2016). *Pterocarpus angolensis* is a leguminous tree, native to the dry woodlands of tropical southern Africa. Its distribution extends from the Northeast of South Africa, Zimbabwe, northern Botswana, and northern Namibia to Angola, Zambia, and Mozambique and reaches its northern limits in southern Kongo and Tanzania (see Supplementary Figure S1). *P. angolensis* tolerates a wide range of environmental conditions and is well adapted to disturbances like fire but rather sensitive to frosts. The climate of the distributional range of *P. angolensis* is characterized by a single wet season and a long dry season. Mean annual rainfall is above 500mm. It grows in diverse soil types, but the tree reaches maximum height on deep, well drained, sandy, or loamy soils (De Cauwer et al., 2014). The distribution of the species is mainly influenced by the amount of summer rainfall, by the minimum temperature in winter and by temperature seasonality. *P. angolensis* is mainly found in areas with annual fire frequency below 45%. Furthermore, the potential distribution of *P. angolensis* is predicted to shrink due to the impacts of climate change especially in the western part of its distributional range (De Cauwer et al., 2014), threatening the future occurrence of the species in Namibia and Botswana.

The species has a high socio-economic and ecological importance since it provides valuable timber, and the sap is used in traditional medicine (Schwartz et al., 2002; Graz, 2004). Forest inventories in Namibia and Southern Angola show that its natural regeneration is limited compared to other woody species (De Cauwer et al., 2018), similar to findings in other parts of southern Africa (Caro et al., 2005). Therefore, it is important to enforce conservation strategies to ensure a sustainable recovery of *P. angolensis* (De Cauwer et al., 2018). However, nitrogen-fixing rhizobia that could improve establishment of the young seedlings have not yet been described for *P. angolensis*; for the Malaysian species *Pterocarpus indicus*, several microsymbionts were described, in order to identify effective strains with potential for application as inoculants in the nurseries and fields (Lok et al., 2006; Lok, 2011).

Therefore, our aim was to study the ability of *P. angolensis* to form nodules with a diverse range of rhizobia and to identify strains which may in future have potential for application as inoculants in nurseries or fields. In order to find potential nodulating strains that are adapted to soils and dry climate, other Southern African shrubs were included in this study. Here, we report the isolation and characterization of *Bradyrhizobium* species that nodulate *P. angolensis* and of symbionts of several other indigenous shrubs of Southern Africa, such as *Wiborgia* and *Indigofera rautanenii*.

## Materials and Methods

### Growth Conditions and Isolation of Bacteria

Bacterial strains were isolated from root nodules of undomesticated legumes grown in different locations of Namibia, Angola, and South Africa (see Supplementary Table S1; Supplementary Figure S1). When available, data on composition of sampled soils are given in Supplementary Table S2. Sampling of root nodules was carried out during the rainy season (Supplementary Table S1). Young *P. angolensis* plants of approximately 20cm height grown in local non-sterile soil in a nursery in Rundu were also examined for nodules.

During transport, nodules were kept in closed glass vials containing dehydrated silica gel (Grönemeyer et al., 2013). Until isolation of bacteria in the laboratory, vials were stored at 4°C. Rehydration and surface sterilization of the nodules and subsequent isolation were carried out as described elsewhere (Grönemeyer et al., 2013). The protocol was adapted to small nodule sizes by carrying out the various wash steps in microcentrifuge tubes and decreasing surface sterilization time in 5% sodium hypochlorite ([v/v] related to Cl) for 0.5 to 1min. Thereafter, nodules were crushed in sterile water; the extract was streaked on modified arabinose gluconate (MAG) agar plates (van Berkum, 1990) and incubated for a minimum of 7days at 28°C. *Bradyrhizobium* strains from type culture collections and own published strains (Grönemeyer et al., 2014, 2017) were grown under the same conditions.

Samples were collected under the Research and collection permit 1757/2012 and 1780/2013 and export permits 90409, 95301, and ES29472, under FLORA 089/2013, FLORA 090/2013, and under contract with ISCED-Huíla with Material Transfer Agreement [11.6.2013 with Ministry of Agriculture, Fisheries & Environment (MINAPA) and ISCED].

### Amplification of the 16S rRNA Gene, 16S-23S rRNA ITS, Housekeeping Genes, and Symbiotic *nodC* Gene

Crude cell lysate of pure bacterial cultures was used as template for PCR amplifications. For lysing cells, colonies from agar plates were resuspended in 100μl of 1 X Tris-EDTA buffer (pH 8) containing 0.1% Tween 20 [vol/vol] and incubated at 95°C for 15min. PCR amplification was performed in 50μl reaction volume containing 2.5U DreamTaq DNA Polymerase (Thermo Fisher Scientific, Waltham, Massachusetts, United States) or MolTaq DNA Polymerase (Molzym, Bremen, Germany), 1 X PCR buffer, 50μM of each deoxyribonucleotide triphosphate (dNTP), 0.5μM of each primer, and 2μl template lysate.

16S rRNA gene amplification was performed using the primer pair Bac8uf and Univ1492r (Edwards et al., 1989; Lane, 1991). The partial 16S-23S rRNA internal transcribed spacer (ITS) region was amplified using the primer pair FGPS1490 and FGPS132’ (Laguerre et al., 1996). Gene fragments of *glnII* and *recA* were obtained with primer pairs glnII12F/glnII689R and recA41F/recA640R, respectively (Vinuesa et al., 2005). For partial *gyrB* amplification, the primer pair gyrB343F/gyrB1043R (Martens et al., 2008) was used with a published protocol (Rivas et al., 2009). Partial sequences of *nodC* were generated with primers NodCfor540/NodCrev1160 (Sarita et al., 2005). Primer sequences and amplification conditions are summarized in Supplementary Table S3.

### Sequencing and Phylogenetic Analyses

Prior to sequencing, PCR products were purified using either the Nucleospin® Gel- and PCR Clean-Up (Macherey-Nagel, Düren, Germany) or the Monarch® Nucleic Acid Purification Kit (NEB, Ipswich, Massachusetts, United States). Sanger Sequencing was carried out by LGC Genomics (Berlin, Germany). For 16S rRNA gene sequences and 16S-23S rRNA ITS region, both DNA strands were sequenced. For shorter protein-coding sequences, only single strand sequencing was performed. All DNA sequences reported in this study were curated for primers and deposited in GenBank, with accession numbers listed in Supplementary Table S3.

Phylogenetic analyses were conducted in MEGA6 (Tamura et al., 2013). Alignments were generated by MUSCLE (16S rRNA, housekeeping marker, and *nodC* gene sequences) and ClustalW2 (ITS sequences) integrated in the MEGA6 software (Edgar, 2004; Larkin et al., 2007). DNA sequences of selected type species and reference strains were retrieved from GenBank. The reference data used in the ITS, MLSA, and *nodC* based phylogenies comprised all *Bradyrhizobium* type strains that were listed in the LPSN database as of July 2019 (Parté, 2014) provided that the sequence was available.

For phylogenetic analyses of ITS sequences, a Neighbor-Joining phylogram was constructed based on the number of nucleotide differences without gap penalty as suggested by Willems (Willems et al., 2001, 2003). For 16S rRNA- and *nodC* gene-based trees, distances were calculated using the Maximum Composite Likelihood method (Tamura et al., 2004). Branch support of the estimated phylogenetic tree was estimated by bootstrapping based on 1,000 pseudoreplicates (Felsenstein, 1985). The MLSA based tree was inferred using the Maximum Likelihood method. The best-fit model of evolution was determined using Model test (Posada and Crandall, 1998). Distances were estimated using the general time-reversible (GTR) model with invariant sites and a gamma distribution of rates across sites. Branch support of the phylogenetic tree was estimated by bootstrapping based on 500 pseudoreplicates (Felsenstein, 1985).

### Characterization of Isolates (PGPR Capabilities)

In order to evaluate the capability of inorganic phosphate solubilization, Pikovskayas Agar plates were used (Gupta et al., 1994). Liquid precultures were grown to an OD_600_ of 0.2–0.3 in MAG medium, and droplets of 10μl were applied to agar plates, 4 per plate, two experiments. Incubation was conducted for a minimum of 1week at 28°C with *Bacillus subtilis* DSM 10 as positive control. The capacity for phosphate solubilization was indicated in this assay by the formation of a white to translucent halo formation around the bacterial colony, rated only weakly positive when it took 8weeks to reach the reaction.

A colorimetric assay was used to indicate the capability for synthesis of indole-3-acetic acid (IAA) from L-tryptophan (Gordon and Weber, 1951). Precultures were grown in 20ml of liquid MAG medium cultures containing 6mM L-tryptophan for 2–5days to an optical density of approximately 1 at 28°C, depending on the growth speed of the strains. Five tenths milliliter of preculture was inoculated to the main culture (medium as above) and grown overnight. After removing cells by centrifugation for 10min, 1ml of supernatant was incubated with Salkowski reagent [0.5M iron (III) chloride in 35% perchloric acid] for 45min in the dark. An absorption peak at 530nm involving a color change from yellow to red indicated the formation of IAA. An absorbance at 530nm of 0.1–0.2 was rated as weak reaction, of around 0.6 as positive reaction. *Azospirillum brasilense* SP7 was used as an IAA-producing positive reference (Ona et al., 2003), and *Azoarcus olearius* BH72 as a negative control. Assays were done in duplicate.

For the detection of siderophore production, a universal assay based on chrome azurol (CAS) and hexadecyltrimethylammonium bromide (HDTMA) bound to iron as indicating complex (Schwyn and Neilands, 1987) was used. Since compounds of the CAS/HDTMA complex may be toxic to cells, the agar plates were overlaid with MAG agar. Liquid precultures were grown to an OD_600_ of 0.2–0.3 in MAG medium, pelleted, and resuspended in SM medium without nitrogen (SM-N, Reinhold et al., 1986) to an optical density of 2; 10μl of the cell suspension were dropped on CAS Agar (four droplets replicated, two experiments), and incubated at 28°C 1–4weeks. The production of siderophores as iron chelators results in the removal of iron from the dye complex and a color change from green to orange as a halo around the colony. *Azospirillum brasilense* Sp7 was used as positive control.

Tests for temperature tolerance were carried out as previously described (Grönemeyer et al., 2014), with maximum growth temperature above 35°C (i.e., growth, at least, at 36°C) regarded as temperature tolerant (Grönemeyer and Reinhold-Hurek, 2018). Fresh overnight precultures in MAG medium grown to OD_600_ of 0.2–0.5 at 28°C were used to inoculate the main cultures with a starting OD_600_ of 0.001 in 20ml of MAG medium. Incubation at 28, 32, 36°C or higher temperature was maintained in a water bath with reciprocal shaking, with three replicate cultures. Cultures were inspected for visible growth within 7days of incubation. A similar set-up was used to analyze NaCl tolerance in 5ml of MAG broth at 28°C with rotary shaking, with 0.5 or 0.75% NaCl added. Moderate salt tolerance was assumed for strains growing with 0.5% NaCl, tolerant strains growing at 0.75% NaCl (Gao et al., 1994; Yao et al., 2015).

### Plant Inoculation and Processing

For inoculation experiments, seeds of domesticated legumes (*Vigna unguiculata*, *Macroptilium atropurpureum*, and *Arachis hypogaea*) and of wild legumes *P. angolensis* and *I. rautanenii* were collected in Namibia. Seeds free of visible damage were surface sterilized with established methods for pulses (Grönemeyer et al., 2014), or with an optimized method for wild legumes. *Pterocarpus angolensis* was treated based on previous experiences (Vander Heyden, 2014); after incubation of pod-released seeds for 15min at 50°C, immersion in 95% ethanol for 2–5min followed by wash in sterile distilled water, further surface sterilization was done for 5–20min in 12% NaOCl (depending on seed size and husk). After five times washing in sterile distilled water, seeds were incubated overnight in sterile water, and then transferred to magenta jars containing a sterile mixture of vermiculite and washed sand (50% each), laced with N-free 0.5 X Jensen’s medium (Bergersen, 1980). Prolonged germination time of the seeds and very poor rates of germination were attempted to be improved, see Results section.

For *I. rautanenii* germination and sterilization, mechanical scratching using sandpaper was followed by immersing seeds for 15min in 95% EtOH, briefly washed with sterile distilled water, and surface-sterilized for 3min in 5% NaOCl followed by five washes in sterile distilled water. Seeds of *Wiborgia tetraptera* were purchased (Sunshine Seeds, Ahlen, Germany), and surface sterilization was extended to 40min in 12% NaOCl. Both were pre-germinated on water agar before transfer to magenta jars.

For bacterial inoculation, strains were grown in liquid MAG medium at 28°C. For qualitative experiments on nodulation capacity, each isolate was assayed in duplicate and all plants were inoculated with 10^9^ cells of the respective isolate in 2ml of 1% (wt/vol) sucrose solution. More plants were used for quantitative assays. In competition assays for nodule occupancy, both individual strains (WR93 and WR4) were mixed in equal proportion according to their optical density representing 2×10^8^ cells per strain per ml, shortly before the plant inoculation. Cells were from exponentially growing cultures (OD_578_ of 0.6–0.9) to ascertain viability. Each plant was inoculated with 1ml of the mixture. In this study, a relatively high titer of bacterial cells was used because of the extended time for tree seedling growth in contrast to pulses, and to mimic inoculant densities applied to field situations. Uninoculated plants were grown as controls. The plants were maintained in a phytotron with day/night (11.5/12.5h) temperatures of 28/25°C at 60% humidity. Plants were watered regularly with sterile distilled water. After 4–12weeks of incubation (as indicated in the results), root systems were examined for the presence of root nodules. Nodule occupancy was evaluated in a PCR-based approach targeting the bacterial ITS sequence inside a respective root nodule. To remove surface bacteria and DNA, root nodules were first subjected to ultrasonication (37kHz for 10min) and then washed three times in 1ml of sterile water. Template DNA was generated by crushing a nodule in 50μl of sterile water. Two microliter of the resulting suspension were used for DNA release followed by PCR amplification and sequencing of the ITS as depicted above. Shoot dry weight was measured after drying for 3days at 65°C.

Fresh weight was determined directly after harvesting for nodule inspection, after gentle blotting with paper towel to remove any free surface moisture; for dry weight determination, plant material was dried at 65°C for 3days.

### Root Nodule Sectioning and Microscopy

After harvesting the root nodules from the legumes, nodules were cut longitudinally with a razor blade and embedded in 4% agarose. After polymerization under vacuum conditions for 15min at room temperature, thin sections of 80–90μm were prepared with a Leica VT1000S Microtome. Sections were incubated in 100-fold diluted SYBR® green I nucleic acid stain (Sigma-Aldrich, Darmstadt, Germany) for at least 30min at 4°C. Morphology of the root nodules was investigated with an Olympus SZX12 stereo microscope equipped with Olympus DF PLAPO 1XPF objective lens (Olympus, Shinjuku, Tokio, Japan) coupled with a Zeiss Axiocam 503 color camera (Zeiss, Oberkochen, Germany).

### Acetylene Reduction Assay

Root systems including the nodules were cut off from shoots, incubated in 10ml tubes sealed with rubber stoppers, and exposed to 10% [v/v] of acetylene for 4h at 30°C. Formation of ethylene was quantified by gas chromatography on an Agilent 7820A GC System, with a column (2m) packed with Shin-Carbon (ST 80/100). Carrier gas was nitrogen with a flow rate of 33ml/min, at 210°C.

### SPAD Value Measurements

Soil-plant analysis development (SPAD) meter values were measured by a SPAD-502 (Minolta Camera Co., Osaka, Japan), 6weeks after inoculation (or uninoculated control plant) and just immediately before the harvest from three uppermost young, expanded leaves of each plant. Three SPAD value readings were taken from a single leaf including one value around the midpoint of leaf blade and the other two values each at approximately 1cm apart from the midpoint. The average of the three readings was considered as the mean SPAD value of each leaf. Means of three leaf data were calculated per plant.

### Statistical Analysis

Treatments were compared by the ANOVA mixed-effects model in GraphPad Prism 9.0, which also takes into account repeated measures in an experiment.

### Tribal Affiliations of the Legumes Used

According to the sub-family classification of the Legume Phylogeny Working Group (LPWG; Azani et al., 2017), most legumes studied here belong to the subfamily Fabaceae-Papilionoideae. The genus *Pterocarpus* is currently assigned to the tribe Dalbergieae, *Indigofera* to the tribe Indigofereae, *Desmodium* to the tribe Desmodieae, *Wiborgia* as well as *Leobordea* to the tribe Crotalarieae. In contrast, *Chamaecrista* belongs to the subfamily Caesalpinioideae in the tribe Cassieae.

## Results and Discussion

### Isolation of Nodule-Associated Bacteria From Legumes of Southern Africa

In previous surveys on Namibian and Angolan pulses (Grönemeyer et al., 2013, 2014), we identified several bradyrhizobial root nodule isolates from Northern Namibia that were heat tolerant (Grönemeyer et al., 2014, 2016; Grönemeyer and Reinhold-Hurek, 2018), including *Bradyrhizobium vignae* which grows even at 40°C. Thus, in drought-prone areas under climate change with higher temperatures, inoculants adapted to these conditions are likely to be of advantage. Therefore, we attempted to find nodulating bacteria for *P. angolensis* in drought-prone woodlands mainly from the respective region, and to cover a broad phylogenetic diversity of potential symbionts. Unfortunately, screening of roots of *P. angolensis* for nodules was not successful (Supplementary Text S1).

Thus, the screening was expanded and shrubs growing in close proximity to *Pterocarpus* in the Kavango were analyzed for symbionts. Isolate WR4^T^ was obtained from root nodules of *I. rautanenii*, a shrub growing close to the Okavango River (Supplementary Table S1) near *Pterocarpus*; it represents the type strain of the novel species *Bradyrhizobium ripae* (Bünger et al., 2018). At the same location, strain WR93 was isolated from *Chamaecrista* sp. root nodule as a single isolate.

Furthermore, legumes from the northern part of the distributional range of *P. angolensis*, the miombo woodlands on the Angolan Central Plateau in Cusseque, Bié Province, Angola, were included in the screen. Miombo woodlands cover much of central and southern Africa and are characterized by the dominance of trees of the Fabaceae subfamily Detarioideae such as *Brachystegia*, *Julbernardia*, and *Isoberlinia*, but also *P. angolensis* occurs here although with lower frequencies than in the Kavango Woodlands. A common subshrub co-occurring with *P. angolensis* is *Desmodium barbatum*. *D. barbatum* is a perennial, erect woody herb or subshrub reaching up to 0.9m. It occurs in grasslands and woodlands in south-central Africa but is also known from South and Central America. According to the IUCN red list (Contu, 2012), it is widespread and rated as “least concern.” *D. barbatum* is locally used for medicine and as forage for livestock. In contrast to inspected *P. angolensis*, its roots exhibited nodules, and strains WR23 and WR27 (carrying identical ITS sequences) were isolated from them (Supplementary Table S1).

The screen was further broadened to undomesticated, wild legume species in other drought-prone areas in Southern Africa. The Kalahari (Pule-Meulenberg et al., 2018) and the Namaqualand of the Northern Cape region, South Africa, are known for long drought periods. For *Wiborgia monoptera*, a highly palatable indigenous shrub of Namaqualand, symbionts are not well described. Isolates WR41 and WR52 were obtained from large, elongated root nodules of two different plants (Supplementary Table S1). In the same location, root nodules were detected from another shrub *Leobordea digitata*, and isolate WR74 was obtained. In the Kalahari of Namibia, *Indigofera alternans* was found to be nodulated, and strain WR96 was isolated (Supplementary Table S1; Supplementary Figure S2).

### Taxonomic Characterization of Nodule-Associated Isolates

The root nodule isolates were first characterized by 16S rRNA sequence analysis in comparison to related species. Isolates of this study, described *Bradyrhizobium* species local to the region and reference strains were included in the trees. Isolate WR95 originating from *P. angolensis* was not affiliated to Alpha- or *Betaproteobacteria*, but was related to *Microbacterium* sp. of the *Actinobacteria*. At 1179 sequence positions (GenBank MK304494), it shared 99.6% sequence identity with the type strain of *Microbacterium aoyamense*. Thus, the isolate was not likely to induce nodules. *Microbacterium* was already previously described as endophytes in nodules of Tunesian legumes (Zakhia et al., 2006).

**Figure 1:**
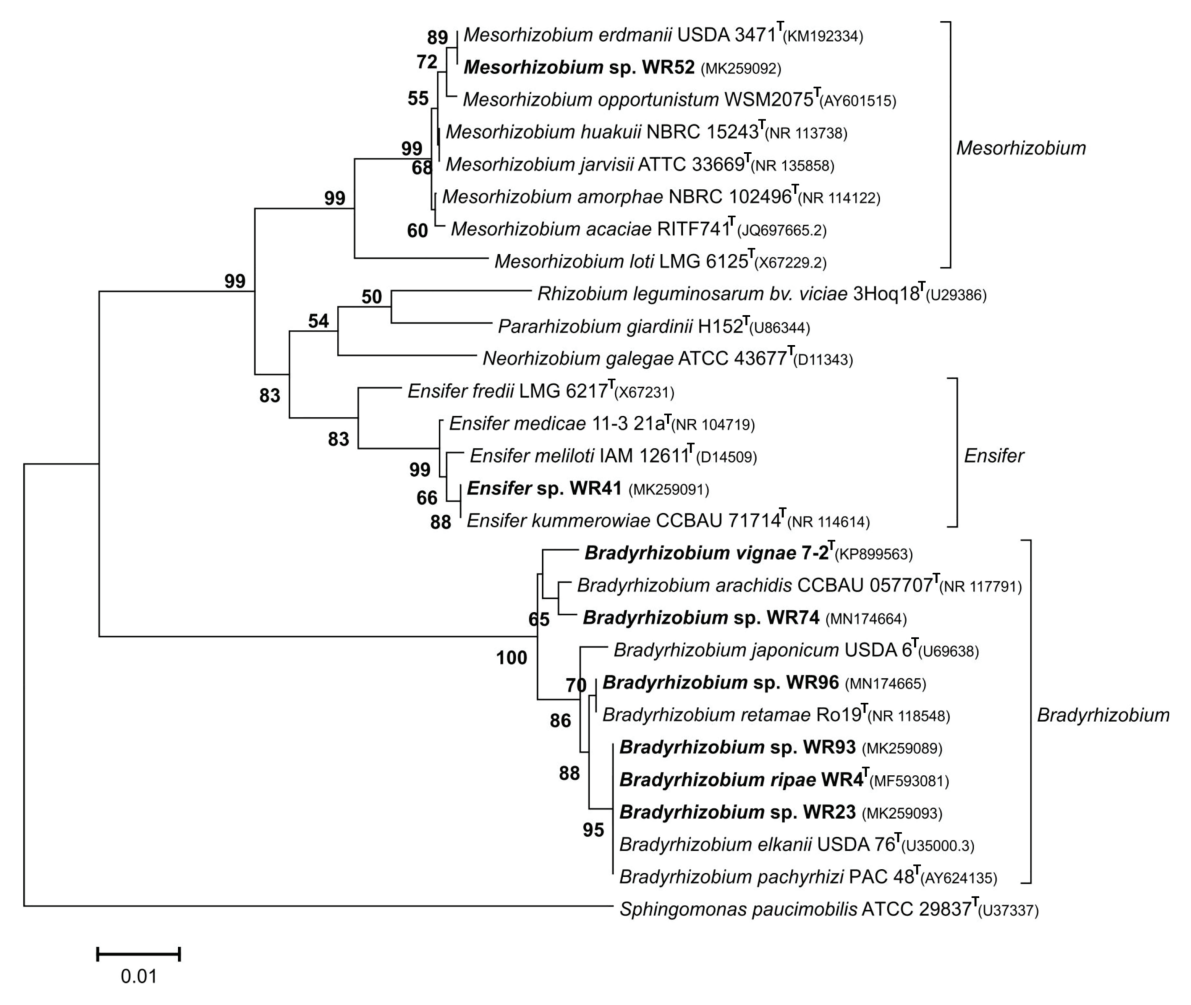
Neighbor-Joining phylogram inferred from partial 16S rRNA gene sequences of isolates and closely related type strains. Type species of selected rhizobial genera were included. Local strains used in the current study are marked in bold. Genbank accession numbers are indicated in parentheses. All strains with species names in the graph are type species. Distances were calculated using the Maximum Composite Likelihood method based on 798 positions. The significance of each branch is indicated by a bootstap value calculated from 1,000 pseudoreplicates (values shown ≥50%). The scale bar indicates the Neighbor-Joining distance.

In contrast, isolates from other indigenous legumes were related to genera of symbiotic rhizobia (Figure 1). Isolate WR41 was identified as *Ensifer* sp. with >99% sequence identity to *Ensifer meliloti* and *Ensifer kummerowiae*, strain WR52 as *Mesorhizobium* sp., both originating from different nodules of *W. monoptera*. To our knowledge, only a single study analyzed the rhizobial community associated with the genus *Wiborgia* except *W. monoptera*, indicating a difference in specificity between *Wiborgia* species depending on their geographical distribution, and a preference for *Rhizobium* and *Mesorhizobium* (Moiloa, 2016). Other isolates, WR23 or WR93 from *D. barbatum* or *Chamaecrista* sp., respectively, and WR74 or WR96 from *Leobordea digitata* or *I. alternans*, respectively, were members of the genus *Bradyrhizobium*.

**Figure 2:**
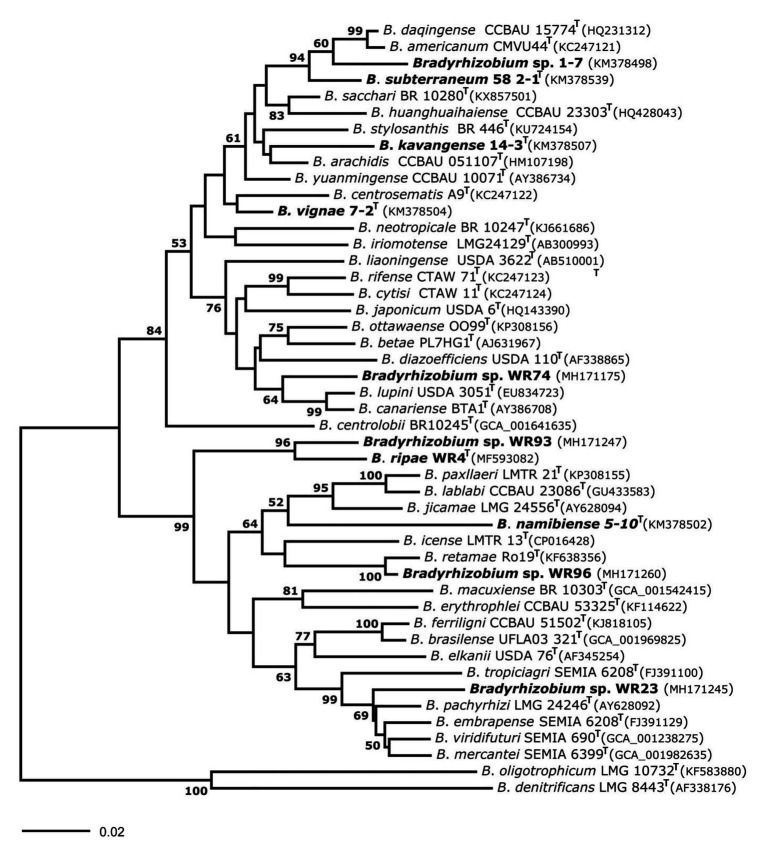
Neighbor-Joining phylogram inferred from partial internal transcribed spacer (ITS) gene sequences of isolates and species-type strains of the genus *Bradyrhizobium*. Isolates and local strains used in the current study are marked in bold. Genbank accession numbers are indicated in parentheses. All strains with species names in the graph are type species. Distances were calculated using the number of differences method based on 1,292 positions without gap penalty. The significance of each branch is indicated by a bootstap value calculated from 500 pseudoreplicates (values shown ≥50%). The evolutionary distances were computed using the *p*-distance method and are in the units of the number of base differences per site (scale bar).

**Figure 3:**
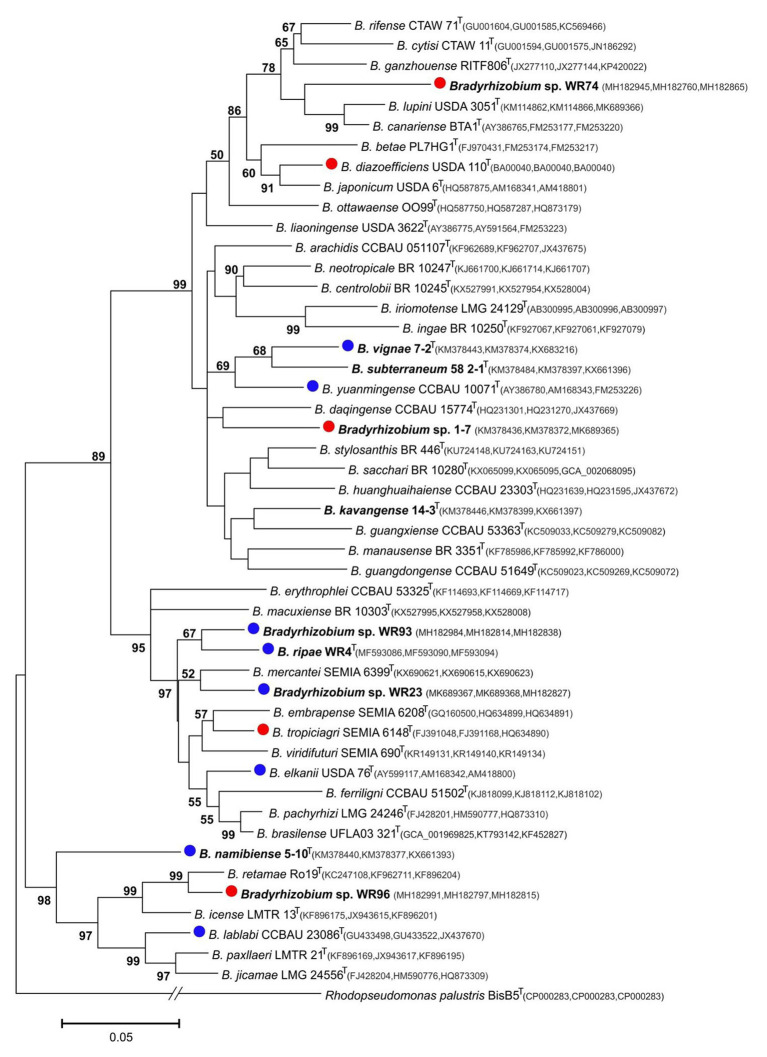
Maximum-likelihood phylogram inferred from concatenated *glnII*-*recA*-*gyrB* sequences of test strains and species type-strains of the genus *Bradyrhizobium*. *Rhodopseudomonas palustris* BisB5 was included as outgroup taxon. Isolates and local strains are marked in bold. Test strains are marked with red (negative for nodulation of *Pterocarpus angolensis*) or blue (positive) dots. Genbank accession numbers are indicated in parentheses. All strains with species names in the graph are type species. Distances were calculated using the Maximum Composite Likelihood method based on 480 positions of *glnII*, 378 positions of *recA* and 548 positions of *gyrB*. The significance of each branch is indicated by a bootstap value calculated from 500 pseudoreplicates (values shown ≥50%). The scale bar indicates the Neighbor-Joining distance.

In the genus *Bradyrhizobium*, the 16S rRNA gene is not a suitable marker for species delineation due to high sequence conservation (van Berkum and Fuhrmann, 2000), and, e.g., strains WR93 and WR23 were not distinguishable from *B. ripae* WR4^T^ and two other *Bradyrhizobium* species (Figure 1). In order to gain more insights into the phylogenetic position of the two *Bradyrhizobium* isolates, intergenic spacer (ITS) sequences were analyzed (Figure 2). They can also be useful to distinguish strains from each other in nodulation competition experiments. All novel isolates were only distantly related to each other, to *B. ripae*, and to other local species *Bradyrhizobium namibiense*, *B. vignae*, *Bradyrhizobium kavangense*, and *Bradyrhizobium subterraneum*. MLSA of several housekeeping genes is used as a reliable method to define phylogenetic relationships and for identification of novel lineages within the genus *Bradyrhizobium* (Rivas et al., 2009). Three concatenated housekeeping genes, *glnII-recA-gyrB*, were used and demonstrated the diversity of the novel strains (Figure 3). Strain WR93 and *B. ripae* WR4^T^ were most closely related to each other albeit at a distance which may suggest assignment to different species (96%). Strain WR23 was distantly related to *Bradyrhizobium mercantei* (96%). Isolates WR74 (92%) or WR96 (97%) were distantly related to *Bradyrhizobium lupini*/*Bradyrhizobium canariense* or *Bradyrhizobium retamae*, respectively, probably also at the rank of species level.

### Putative Plant Growth Promotion Characteristics of Root-Nodule Associated Strains

Even though non-rhizobial strains may not be able to induce root nodules, as endophytes in nodules, they may have a beneficial effect on plant growth when co-inoculated with rhizobial strains (Masciarelli et al., 2014; Tariq et al., 2014; Korir et al., 2017). In contrast, some strains may be of opportunistic nature, exploiting the nutrient-rich nodule environment induced by rhizobia (Ibànez et al., 2009). Therefore, we analyzed PGP characteristics and also the host range for pulses of the nodule-associated isolates (Table 1).

**Table 1:** Plant-associated characteristics of analyzed rhizobial and non-rhizobial strains.

Strain^1^	Host plant	Country of origin	Distinctive characteristics of the strains									
			1^2^	2	3	4	5	6	7	8	9	10
*Bradyrhizobium vignae* 7-2^T^	*Vigna unguiculata*	NAM^3^	−^4^	−	−	+	+	nd	+	41°C	±	+
*Bradyrhizobium ripae* WR4^T^	*Indigofera rautanenii*	NAM	−	nd	+	+	+	nd	*	36°C	++	+
*Bradyrhizobium* sp. WR23	*Desmodium barbatum*	ANG	+	−	±	+	+	nd	*	nd	±	+
*Bradyrhizobium* sp. WR93	*Chamaecrista* sp.	NAM	−	−	+	+	nd	*	*	28°C	±	+
*Bradyrhizobium* sp. WR74	*Leobordea digitata*	SA	+	−	±	±	*	nd	*	28°C	−	−
*Bradyrhizobium* sp. WR96	*Indigofera alternans*	NAM	−	−	−	+	++	nd	+	36°C	±	−
*Ensifer* sp. WR41	*Wiborgia monoptera*	SA	−	+	+	+	nd	+	nd	nd	nd	−
*Mesorhizobium loti* WR52	*Wiborgia monoptera*	SA	+	−	−	+	nd	+	nd	nd	nd	−
*Microbacterium* sp. WR95	*Pterocarpus angolensis*	NAM	−	−	±	−	−	−	nd	nd	nd	−

1 References for strain origins are given in Supplementary Table S1.

2 Distinctive characteristics of the strains: 1, phosphate solubilization; 2, production of siderophores; 3, putative IAA production; 4, *nodC* gene amplification; 5, *Vigna unguiculata* nodulation; 6, *Macroptilium atropurpureum* nodulation; 7, *Arachis hypogaea* nodulation; 8, temperature tolerance as maximum growth temperature in MAG medium; 9, NaCl tolerance in MAG medium, at 0.5% (±) or 0.75% (++) NaCl; 10, *Pterocarpus angolensis* nodulation.

3 NAM, Namibia; ANG, Angola; SA, South Africa.

4 ++, strongly positive; +, positive; −, negative; ±, weakly positive; nd, not determined; *, stunted growth/ineffective nodulation.

Phosphorus is a major macronutrient essential for metabolic energy processes. Especially for legumes, a phosphate deficiency is a critical limitation in ecosystems since important processes responsible for symbiotic N_2_ fixation require phosphate (Kleinert et al., 2014). Depending on soil type and composition, concentrations of soluble phosphorus can be low and the supply for the plant insufficient (Gyaneshwar et al., 2002). Microorganisms able to solubilize mineral phosphate through secretion of organic acids may improve nutrient supply to legume plants. Strains *Mesorhizobium* sp. WR52 and *Bradyrhizobium* sp. WR23 as well as WR74 were able to solubilize phosphate (Table 1).

Like phosphate, iron deficiency may lead to limitation of nodule development and nitrogen fixation. Due to involvement of iron in several key proteins like nitrogenase or the protective leghaemoglobin, an effective symbiosis may not be established (O’Hara et al., 1988). Production of siderophores on overlaid chrome azurol S (CAS) plates was shown only by *Ensifer* WR41.

Besides the improvement of plant nutrient composition, also the production of plant hormones can alter plant growth and development. The production of the auxin IAA stimulates the formation of lateral roots, leading to an increased number of sites for rhizobial interaction (Spaepen et al., 2007). In culture, *Ensifer* WR41, *Bradyrhizobium* sp. WR93, and *B. ripae* WR4^T^ showed a positive reaction in a colorimetric assay for IAA production from L-tryptophan. A weak reaction was also observed for *Microbacterium* WR95 and *Bradyrhizobium* spp. WR74 and WR23. Hints for synthesis of indole-related compounds, such as IAA and its precursors indole-acetamide (IAM) or indole-3-pyruvic acid (IPA) are detectable in such assay (Gilbert et al., 2018).

As previously described (Grönemeyer et al., 2016), *B. vignae* 7-2^T^ is highly temperature tolerant; also strains WR4^T^ and WR96 were quite tolerant (36°C), while strains WR93 and WR74 grew only at 28°C. NaCl tolerance, however, was high for *B. ripae* WR4^T^. All *Bradyrhizobium* strains induced nodules on at least one of the tested pulses cowpea, peanut, or *Macroptilium* (Table 1). In contrast, *Microbacterium* sp. WR95 did neither induce nodules on any of these plants, nor was it positive for amplification of the *nodC* gene, indicating an endophytic rather than a nodule-symbiont lifestyle.

### Evaluation of Nodulation of *Pterocarpus angolensis* and Undomesticated Legume Shrubs by Indigenous Rhizobia

Only few studies described microscopic nodule structures of endemic Southern African legumes (Sprent et al., 2013; Lemaire et al., 2015a). In particular, undomesticated legumes inoculated with indigenous rhizobia have been poorly investigated so far. In order to authenticate nodule isolates and to define potential inoculant for *P. angolensis*, inoculation was carried out under sterile conditions in vermiculite. However, germination of seeds occurred at variable times (3–8weeks) after surface sterilization and at very poor rates (8–10%). Therefore, we attempted to improve the process. Clipping of the seeds after surface sterilization enhanced germination to 33% within 1–2weeks. Another improvement was the timing of inoculation: inoculating germinated seedlings at early two leaf stage often arrested their further growth. Inoculation after the emergence of the small third budding leaf was mostly successful and further growth continued.

The putative *Pterocarpus* endophyte *Microbacterium* sp. WR95 did not induce any root nodules as expected (not shown). In previous studies on root nodules of *Pterocarpus erinaceus* and *Pterocarpus lucens* native of sudanean and sahelian regions of Senegal, fast growing *Rhizobium* and *Mesorhizobium* strains as well as slow growing bacteria related to *Bradyrhizobium elkanii* and *Bradyrhizobium japonicum* were isolated (Sylla et al., 2002). *Pterocarpus indicus* was found to be effectively nodulated by *B. elkanii*; however, *Mesorhizobium ciceri*, *Sinorhizobium meliloti*, and *Rhizobium* spp. induced ineffective nodules (Lok et al., 2006). Therefore, a wide taxonomic range of rhizobia had to be tested to find potential symbionts for *P. angolensis*.

**Figure 4:**
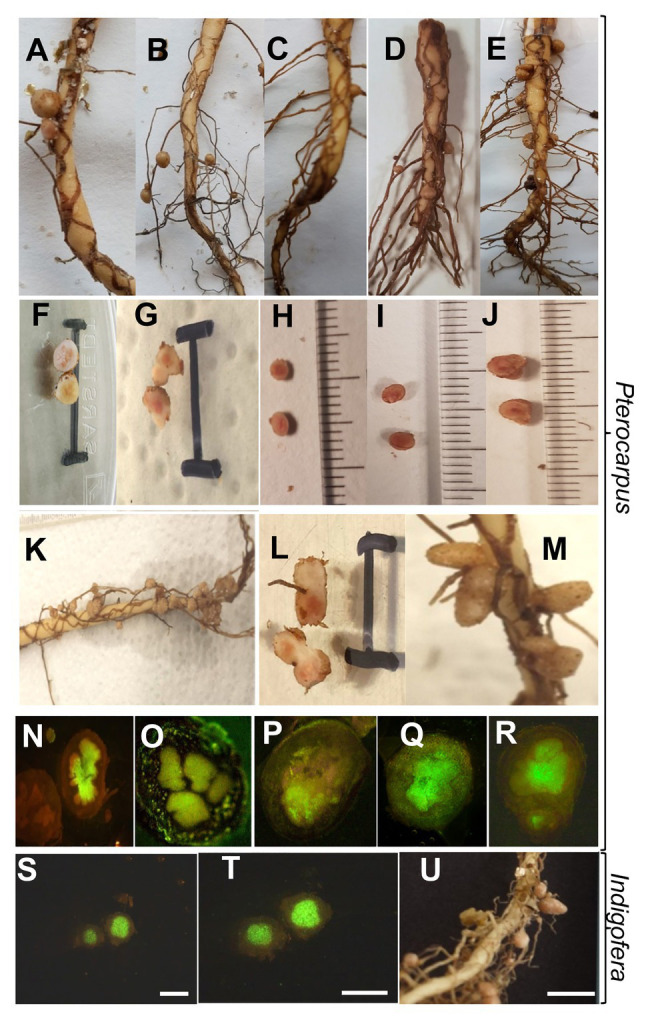
Morphology of nodules of *P. angolensis* **(A–R)** and *Indigofera rautanenii* **(S–U)** induced by *Bradyrhizobium* spp. isolates. Overview of nodules on main root **(A,C–D,K,M)** or lateral root **(B)** and dissected single nodules **(F–J,L)** with scale of 10mm. **(N–T)**, Fluorescence micrographs of longitudinal thin sections through nodules stained with SYBR green nucleic acid stain. Rhizobia-infected regions appear green, bars indicate 1mm. Plants were grown in sterile vermiculite/sand mixture inoculated with *Bradyrhizobium* sp. WR93 **(A,F,N)**, *Bradyrhizobium ripae* WR4^T^ **(B,G,O,S–U)**, *Bradyrhizobium elkanii* LMG 6134^T^ (= USDA76^T^; **C,H,P**), *Bradyrhizobium namibiense* 5-10^T^ **(D,I,Q)**, *Bradyrhizobium yuanmingense* LMG 21827^T^ **(E,J,R)**, *Bradyrhizobium* sp. WR23 **(K,L)**, or *Bradyrhizobium vignae* 7-2^T^ **(M)**. For *P. angolensis* or *I. rautanenii*, 6 or 12weeks post inoculation, respectively.

Southern African isolates of shrubs *Mesorhizobium* sp. WR52 or *Ensifer* sp. WR41 did not induce any nodules in *P. angolensis* (not shown). Thus, we focused on members of *Bradyrhizobium*. Among the isolates of this study, *Bradyrhizobium* sp. WR93 (Figures 4A,F) and WR23 (Figures 4K,L), and *B. ripae* WR4^T^ (Figures 4B,G) induced pink, putatively efficient root nodules; although none of these strains originated from this tree, they all emanated from nodulated plants growing close to *P. angolensis*. In a competition assay using ITS sequences to differentiate between strains occupying the nodules, carried out on three plants co-inoculated with similar numbers of bacteria, *Bradyrhizobium* sp. strain WR93 appeared to outcompete *B. ripae* strain WR4^T^ for nodulation of *P. angolensis* (roughly 6:1 nodules occupied).

As *Bradyrhizobium* species are often promiscuous, we, furthermore, tested several novel species that originated from the Namibian Kavango region as well, those from nodules of cultivated pulses. The heat-tolerant *B. vignae* 7-2^T^ from cowpea (Figure 4M; Grönemeyer et al., 2016) as well as *B. namibiense* 5-10^T^ from Lablab (Figures 4D,I; Grönemeyer et al., 2017) induced nodules, while an unnamed strain from peanut *Bradyrhizobium* sp. 1–7 (Grönemeyer et al., 2014) did not (not shown).

This indicated that surprisingly, strains of both phylogenetic lineages, *B. japonicum* and *B. elkanii*, can induce root nodules on this tree, suggesting high promiscuity. Therefore, we tested several other reference species of both lineages. *Bradyrhizobium diazoefficiens* USDA 110^T^ and *B. tropiciagri* SEMIA 6148^T^ did not induce nodules, while members from both different lineages *B. elkanii* LMG 6134^T^ (Figures 4C,H), *B. lablabi* CCBAU 23086^T^ (not shown), and *B. yuanmingense* LMG 21827^T^ (Figures 4E,J) nodulated; *B. elkanii* was also described in previous studies as symbiont on other *Pterocarpus* species (Sylla et al., 2002; Lok et al., 2006). Although both, *B. diazoefficiens* USDA 110^T^ and *B. elkanii* USDA 76^T^ (= *B. elkanii* LMG 6134^T^), are microsymbionts for the same host soybean, they differ in various traits such as DNA fingerprint, rhizobitoxine production, IAA production, and uptake of hydrogenase. The differences between both species might extend to host preference in some cases. Indeed, *B. japonicum* strain USDA 110^T^ fell into *nod* group A, while *B. elkanii* strain USDA76^T^ fell into *nod* group B based on *nod* RFLP patterns (Minamisawa et al., 1997). Accordingly, the two-way signaling mediated by flavonoids and lipo-chitin nodulation signals is not involved in the host preference of *B. japonicum* and *B. elkanii* within soybean bradyrhizobia. Due to such fine differences in the *nod* groups even between the strains of two different species, it might be plausible that soybean microsymbiont strain *B. diazoefficiens* USDA 110^T^ does not nodulate *P. angolensis* while *B. elkanii* USDA 76 ^T^ does so.

**Figure 5:**
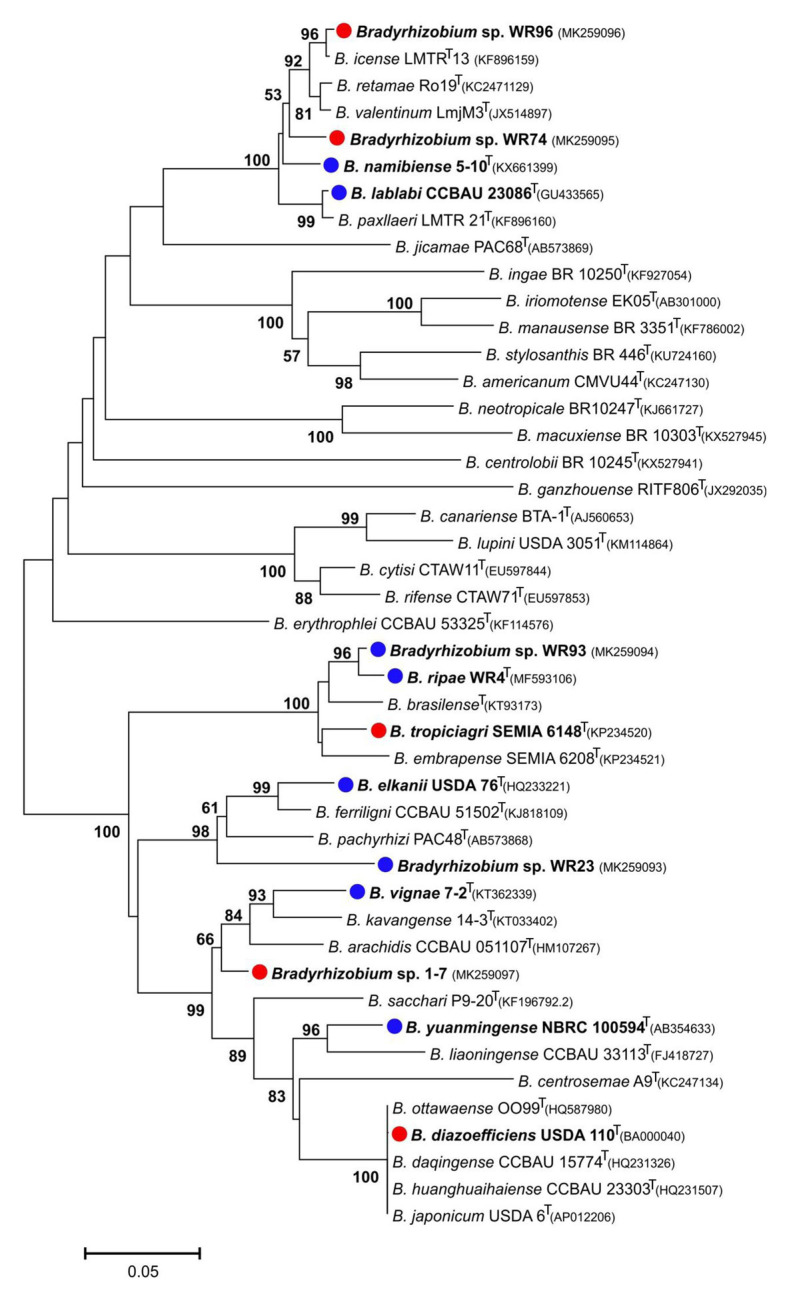
Neighbor-Joining phylogram inferred from partial *nodC* gene sequences of test strains and species-type strains of the genus *Bradyrhizobium*. Test strains are marked with red (negative for nodulation of *P. angolensis*) or blue (positive) dots. Genbank accession numbers are indicated in parentheses. All strains with species names in the graph are type species. Distances were calculated using the Maximum Composite Likelihood method based on 426 positions. The significance of each branch is indicated by a bootstrap value calculated from 1,000 pseudoreplicates (values shown ≥50%). The scale bar indicates the Neighbor-Joining distance.

Given that *Bradyrhizobium* tends to be a dominant, diverse rhizobial genus among rhizobia in Namibian soils (Grönemeyer et al., 2014; Grönemeyer and Reinhold-Hurek, 2018), we found promising, environmentally adapted strains effectively nodulating *P. angolensis*, with potential as inoculant strains for nursery stations. The analysis of symbiosis-related genes might aid in defining symbiovars of rhizobial strains. As the nodulating bacterial strains originated from different hosts and were phylogenetically placed in different lineages, *nodC* gene sequences that are related to Nod-factor production were generated and compared (Figure 5). Indeed, two *P. angolensis* symbionts WR4^T^ and WR93 had highly related *nodC* gene sequences; however, genes of the other symbionts WR23 and *B. vignae* 7-2^T^ as well as other nodulating and non-nodulating type strains were scattered across the phylogenetic tree (Figure 5). Again, a large phylogenetic distance of strains nodulating *Pterocarpus* was evident, as they belonged to two different *nodC* clades III and IV according to the nomenclature of Degefu (Degefu et al., 2018). This indicates that *P. angolensis* might be quite promiscuous with respect to symbiovars.

Occurrence of *nod* genes and root nodule induction of rhizobia are not the sole essential criterion for selection of successful inoculant strains. It is important that the nodulating bradyrhizobia actually fix nitrogen in nodule tissue and thereby improve growth of *P. angolensis* under controlled conditions. Thus, several of the *Pterocarpus* nodulating strains were individually inoculated onto germinated *Pterocarpus* seedlings under aseptic conditions in vermiculite:sand mixture in at least two biological independent experiments. After 6 weeks of incubation in the phytotron, the inoculated seedlings harbored much darker, green leaves with broad leaf blades in contrast to the non-inoculated ones (Supplementary Figure S3A). To quantify this effect, SPAD values were measured as an indication of chlorophyll fluorescence. The average SPAD values of the three uppermost fully expanded leaves of inoculated plants were higher than those of the uninoculated ones (Supplementary Figure S3B). This indicated improved nitrogen supply by the inoculant.

As mentioned earlier, the plants inoculated with positive candidate strains were always nodulated (Figures 4A–E). Dissected nodule sections appeared pink in the middle, attributing to leghaemoglobin, an indication of active nitrogen fixation (Figures 4F–J). The uninoculated plants were always devoid of any nodules. The determinate nodules varied in size, number, and distribution on the tap root or lateral roots depending on the symbiont (Figures 4A–E). In all cases, one or two nodules from individual inoculation were used for the re-isolation and identification of the nodulating rhizobia by sequence analysis. Always, the same rhizobial strain was recovered from the nodule tissue that was used for the original inoculation.

**Figure 6:**
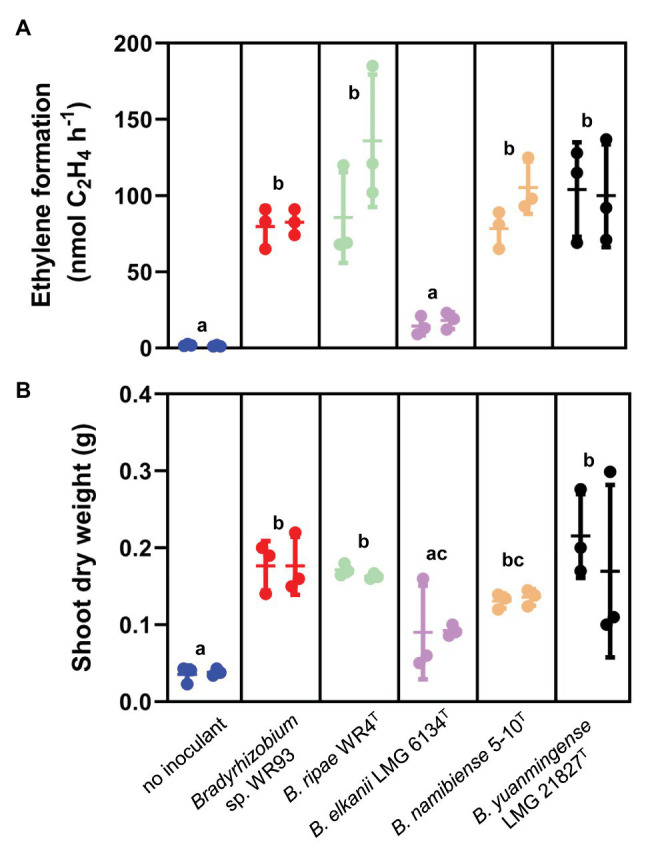
Active nitrogen fixation of root nodules **(A)** and improved growth of the shoots **(B)** of *P. angolensis*, induced by respective isolates of *Bradyrhizobium* spp. indicated under each block. Plants were grown in sterile vermiculite/sand mixture inoculated with the respective *Bradyrhizobium* strain under aseptic conditions and harvested 6weeks after inoculation; non-inoculated plants (no nodules induced) as negative control. **(A)** Nitrogenase activity measured by acetylene reduction assay (ARA) from nodulated root systems, given as ethylene accumulation (nmol of ethylene produced per hour per root system). **(B)** Shoot dry weight from the same experiment as **(A)**. Data from two independent inoculation experiments with three plants each. Scatter dot plots shown with means (horizontal line), SD (bars), and original values as dots for both experiments. Data of treatments with different letters indicate statistical significance (*p*<0.05) using an ANOVA mixed-effects model (GraphPad Prism 9.0).

Active nitrogen fixation of the root nodules was further substantiated by acetylene reduction assay measured by gas chromatography. All tested strains led to significant acetylene reduction in comparison to uninoculated control plants, except for *B. elkanii* LMG 6134^T^ for which ethylene formation was low and not significantly different from controls (Figure 6A). Although *P. angolensis* – as a tree – grew very slowly and has rather large *N*-containing seeds, positive effects on plant growth were already observed after this short time. Parallel to active root nodule nitrogen fixation, almost all *P. angolensis* seedlings inoculated with diverse rhizobial lineages experienced a boost in the shoot biomass (Figure 6B), with significantly increased fresh weights (Supplementary Figure S3C) and dry weights in comparison to shoots of uninoculated controls. Again, *B. elkanii* LMG 6134^T^ was an exception, as only low, highly variable increase of shoot dry weight was obtained, and the symbiosis was apparently not very effective at this stage. It can be concluded that the *Pteroocarpus* nodules formed by a diverse range of rhizobia tested are capable to (1) actually fix nitrogen and (2) support growth and nitrogen content of the host plant, *P. angolensis*. Although no report about the promiscuity of *P. angolensis* is known, it was already suggested (Sprent and Parsons, 2000) that nodulating species of *Pterocarpus* such as *P. indicus* (related species) are promiscuous tree legumes. This was further substantiated by studies on Malaysian *P. indicus* (Lok et al., 2006; Lok, 2011) which demonstrated nodulation by diverse strains of rhizobia from four genera, *Bradyrhizobium*, *Rhizobium*, *Sinorhizobium*, and *Mesorhizobium*. Therefore, *P. indicus* appears to be also a promiscuous host for nodulation.

**Figure 7:**
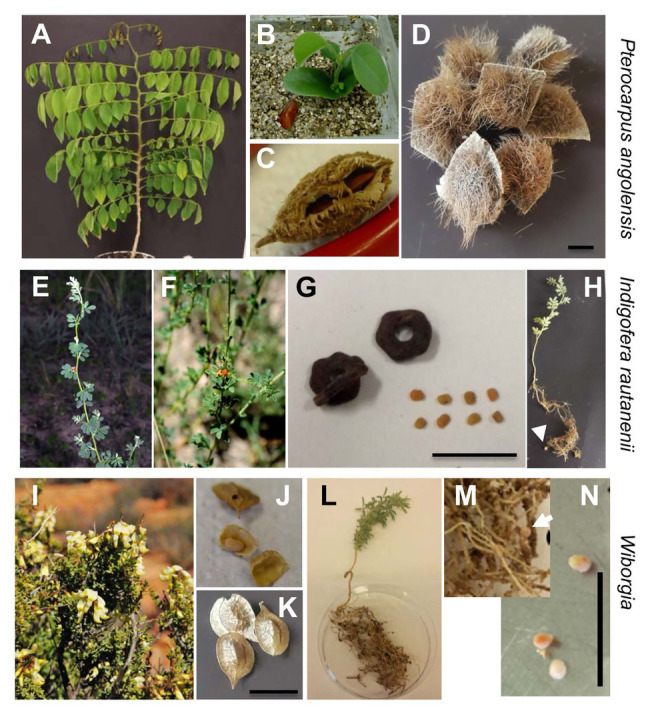
Indigenous legumes with respective seeds, seedlings, and nodules. **(A–D)** *Pterocarpus angolensis*, **(E–H)** *I. rautanenii*, **(I)** *Wiborgia monoptera*, **(J–N)** *Wiborgia tetraptera*. Plants grown in Kavango region, Namibia **(E,F)** or Namaqualand, South Africa **(I)**. Collected pods and seeds **(C,D,G,J,K)** and young plants grown in the phytotron from them **(A,B,H,L,M)**. Nodules after inoculation with *B. ripae* WR4^T^ **(H)** and *Mesorhizobium loti* WR52 40days post inoculation (**M,N** dissected). Arrows point to nodules. Bars indicate 1cm.

In order to authenticate our isolates for their other original indigenous hosts, we carried out further nodulation tests under sterile conditions whenever possible with respect to seed availability and germination. The perennial legume *I. rautanenii* is indigenous to Southern Africa, mainly Namibia (Van Wyk and Gericke, 2000), and to our knowledge, there are no studies on the rhizobial diversity of this species. Various other *Indigofera* species were investigated with respect to rhizobial diversity and pointed out that strains of the genus *Bradyrhizobium* are some of the most common symbionts (Aserse et al., 2012; Lemaire et al., 2015a; Reeve et al., 2015). Accordingly, strain *B. ripae* WR4^T^ originally isolated from *I. rautanenii* could induce effective nodules on this species (Figures 4, 7). However, the genus *Indigofera* appears to be quite promiscuous; it was reported (Lemaire et al., 2015b) that six *Indigofera* species were associated with a variety of symbionts, with four distinct groups of beta- (*Burkholderia*) and alpha-rhizobia (*Bradyrhizobium*, *Ensifer*, and *Mesorhizobium*), and is the most promiscuous legume group in the Fynbos.

Seeds of the South African species *W. monoptera* were not available. In order to test nodulation by isolates from this species, we used seeds from another species of the same genus, *W. tetraptera*, which could be commercially purchased. Isolate *Mesorhizobium* sp. WR52 induced effective nodules, and within 40days, 20–40 nodules per plant were obtained (Figure 7). Isolate *Ensifer* sp. WR41 did not induce nodules on this species. For *D. barbatum*, seeds were unfortunately not available, thus the isolate was not tested on the original host. However, for both strains, we analyzed the *nodC* sequence as a marker for symbiotic genes. *Mesorhizobium* sp. WR52 *nodC* clustered with a clade of mesorhizobia of unknown species affiliation (Supplementary Figure S4). All these strains were isolated from South African Cape Fynbos, most of them from *Aspalathus* species (Lemaire et al., 2015b) which belong to the tribe Crotalarieae like *Wiborgia*; strains might thus belong to symbiovars of Fynbos shrubs. Surprisingly, *nodC* of *Ensifer* sp. WR41 did not cluster within *Ensifer* genes, but with our *Mesorhizobium nodC* (Supplementary Figure S4), albeit with low bootstrap support. Lateral gene transfer of symbiosis genes appears to be quite common in the Fynbos biome (Lemaire et al., 2015b). Also in other phylogenetic *nodC* analyses, not all *Ensifer* and *Mesorhizobium* genes appeared to be monophyletic for the genus (Laguerre et al., 2001; Rejili et al., 2020), and horizontal transfer of symbiosis genes can even occur between different rhizobial genera (Andrews et al., 2018).

For two selected endemic legumes species, nodule structures were evaluated. Legumes inoculated under sterile conditions as above were grown in vermiculite, and nodule sections were investigated under the microscope (Figure 4). As alternative to toluidine blue staining, root sections were stained with SYBR green nucleic acid stain to highlight the bacteroidal zone based on staining of highly concentrated bacterial DNA. Nodules showed densely colonized central zones (Figure 4), typical for dalbergioid nodules, in which the central tissue is uniformly infected (Lavin et al., 2001), and infection by crack entry through lateral root junctions is common (Coba de la Pena et al., 2017). Also, *Pterocarpus* is within the dalbergoid clade as part of the *Pterocarpus* clade (Lavin et al., 2001). In general, nodules of *P. angolensis* had an average diameter of 0.3–0.45cm with a spherical to elongate, determinate shape and were mainly found on the strong main root after growth for 6weeks (Figure 4). For *I. rautanenii*, mature nodules in our study were of indigoferoid, indeterminate shape (Figure 4), whereas young nodules appeared round. On average, mature nodules sampled were 0.25cm in length and 0.15cm in width with a central pink (leghemoglobin) pigmented core.

## Concluding Remarks

Earlier studies have shown that some tree species occurring in Miombo and other woodlands, such as *Pterocarpus angolensis* or *Pericopsis angolensis*, are difficult to grow in nurseries and that problems arise at several stages: germination, seedling survival, and seedling establishment (Chidumayo, 2002; Vander Heyden, 2014). However, rhizobial inoculant technology may overcome some of these difficulties, as nitrogen-fixing symbionts may provide nitrogen in N-poor soils and thereby improve seedling vigor. Additional putative plant growth-promoting characteristics of the inoculants may further foster seedling growth. Thus, they may not only effectively nodulate but also improve Pterocarpus plant growth by other means. In conclusion, we have isolated and identified symbionts of *P. angolensis* that might be a first step into developing inoculants to be used in nurseries or in assisted forest regeneration for *P. angolensis*, a valuable timber source.

## Data Availability Statement

The datasets presented in this study can be found in online repositories. The names of the repository/repositories and accession number(s) can be found in the article/Supplementary Material.

## Author Contributions

BR-H, WB, AS, and TH planned the experiments. BR-H, TH, and RR carried out sampling in Africa. AS isolated the bacteria. WB, AS, JZ, and JG characterized the isolates. WB and AS carried out the plant experiments. WB, AS, and BR-H wrote the manuscript with editing remarks of all other authors. All authors contributed to the article and approved the submitted version.

### Conflict of Interest

The authors declare that the research was conducted in the absence of any commercial or financial relationships that could be construed as a potential conflict of interest.
