# Supplementary material for: Root Nodule Rhizobia From Undomesticated Shrubs of the Dry Woodlands of Southern Africa Can Nodulate Angolan Teak Pterocarpus angolensis, an Important Source of Timber
Source: Front Microbiol. 2021 Jan 28;12:611704. doi: 10.3389/fmicb.2021.611704 (PMC7876412; doi:10.3389/fmicb.2021.611704)
Supplement: Supplementary file 1 [file Data_Sheet_1.pdf]

Supplementary Material

Root nodule rhizobia from undomesticated shrubs of the dry Woodlands of Southern Africa can nodulate Angolan teak *Pterocarpus angolensis*, an important source of Timber

Wiebke Bünger[a], Abhijit Sarkar[a], Jann Lasse Grönemeyer[a], Janina Zielinski[a], Rasmus Revermann[b], Thomas Hurek[a] and Barbara Reinhold-Hurek[a,]*

[a] Department of Microbe-Plant Interactions, CBIB (Center for Biomolecular Interactions), Faculty Biology and Chemistry, University of Bremen, D-28359 Bremen, Germany;

[b] Department Biodiversity, Ecology and Evolution of Plants, Institute for Plant Science and Microbiology, University of Hamburg, D-22609 Hamburg, Germany.

Supplementary Text S1. Screening of *Pterocarpus angolensis* for nodules.

Sites in Northern Namibia close to Rundu near the Okavango river were selected where large trees of *P. angolensis* were growing. However, nodules were not detected on inspected unearthed roots of *P. angolensis* trees (four) which would have been the prime source for symbionts, and small tree seedlings were not found at the inspected sites. Even in the rainy season, unearthing deep roots from Kavango soils without detaching putative nodules was difficult. Therefore, from a nursery in Rundu, Namibia, roots of young *P. angolensis* plants were screened for root nodules; however, among four plants no convincing nodule structures were detected. Only one root showed slight protrusions that were nevertheless used for cultivation of rhizobia. The fast-growing strain WR95 was isolated from these surface-sterilized structures (**Supplementary Table S1**). Also among seedlings grown for 7 weeks in a germination experiment in small pots containing sand-compost mixtures in Namibia (De Cauwer et al., 2018), approximately 20 inspected seedlings did not show nodules.

De Cauwer, V., Chaka, M., Chimwamurombe, P.M., George, D., Ham, H., Heita, H., et al. (2018). "Artificial and assisted natural regeneration of socio-economically important southern African tree species," in *Climate change and adaptive land management in southern Africa – assessments, changes, challenges, and solutions,* eds. R. Revermann, K.M. Krewenka, U. Schmiedel, J.M. Olwoch, J. Helmschrot & N. Jürgens. (Göttingen, Windhoek: Klaus Hess Publishers), 324-331.

Table S1│ Origin of bacterial isolates

Strain name	Host plant	Sampling date for novel isolates	Location/Coordinates	Reference
Bradyrhizobium vignae 7-2^T	*Vigna unguiculata* (L.)Walp.		1. Namibia, Mashare, MADI Res. Station S17.892806, E20.210467	(Grönemeyer et al., 2016)
Bradyrhizobium ripae WR4^T	*Indigofera rautanenii* Baker f.		2. Namibia, Nkwazi Lodge, near Okavango river S17.865515, E19.908073	(Bünger et al., 2018)
Bradyrhizobium namibiense 5-10^T	*Lablab purpureus* (L.) Sweet		3. Namibia, Mashare, MADI Res. Station S17.89274, E20.21068	(Grönemeyer et al., 2017)
Bradyrhizobium sp. 1-7	*Arachis hypogaea* L.		4. Namibia, Mashare, MADI Res. Station S17.895486, E20.211047	(Grönemeyer et al., 2014)
Bradyrhizobium sp. WR23, WR27	*Desmodium barbatum* (L.) Benth.	March 2013	5. Angola, Bié Province, Miombo woodlands near Cusseque S13.69972, E17.06894	This study
Ensifer sp. WR41	*Wiborgia monoptera* E. Mey.	October 2013	6. South Africa, Namaqualand S30.385722, E 18.28516	This study
Mesorhizobium sp. WR52	*Wiborgia monoptera* E. Mey.	October 2013	6. South Africa, Namaqualand S30.385722, E 18.28516	This study
Bradyrhizobium sp. WR74	*Leobordea digitata* (Harv.) B.-E.	October 2013	7. South Africa, Namaqualand S30.385722, E 18.28517	This study
Bradyrhizobium sp. WR93	*Chamaecrista* sp.	February 2014	2. Namibia, Nkwazi Lodge S17.865515, E19.908073	This study
Microbacterium sp. WR95	*Pterocarpus angolensis* DC.	February 2014	8. Namibia, Nursery in Rundu -S17.9333, E19.7667	This study
Bradyrhizobium sp. WR96	*Indigofera alternans* DC.	February 2014	9. Namibia, Kalahari near street Gobabis-Drimiopsis, S22.290000, E018.99632	This study

Bünger, W., Grönemeyer, J.L., Sarkar, A., and Reinhold-Hurek, B. (2018). *Bradyrhizobium ripae* sp. nov., a nitrogen-fixing symbiont isolated from nodules of wild legumes in Namibia. *Int. J. Syst. Evol. Microbiol.* 68**,** DOI 10.1099/ijsem.1090.002955.

Grönemeyer, J.L., Bünger, W., and Reinhold-Hurek, B. (2017). *Bradyrhizobium namibiense* sp. nov., a symbiotic nitrogen-fixing bacterium from root nodules of *Lablab purpureus*, hyacinth bean, in Namibia. *Int. J. Syst. Evol. Microbiol.* 67**,** 4884-4891.

Grönemeyer, J.L., Hurek, T., Bünger, W., and Reinhold-Hurek, B. (2016). *Bradyrhizobium vignae* sp. nov., a nitrogen-fixing symbiont isolated from effective nodules of *Vigna* and *Arachis*. *Int. J. Syst. Evol. Microbiol.* 66**,** 62-69.

Grönemeyer, J.L., Kulkarni, A., Berkelmann, D., Hurek, T., and Reinhold-Hurek, B. (2014). Identification and characterization of rhizobia indigenous to the Okavango region in Sub-Saharan Africa. *Appl. Environ. Microbiol.* 80**,** 7244-7257.

Table S2 │ Soil data for sampling location of isolates

Strain name	Location	pH in H_2O [a]	EC in 1:2.5 soil-water [µS cm-1]	Total organic carbon [%]	C/N ratio	Plant available Phosphorous [mg kg^{-1}]	Plant available potassium [g kg^{-1}]
Bradyrhizobium vignae 7-2^T	1. Namibia, Mashare[b]	7.6	96	0.42	8.6	35.4	0,174
Bradyrhizobium ripae WR4^T	2. Namibia, Nkwazi Lodge, near Okavango river S17.515515, E19.542997	5.4	36	1.37	11.6	0.057	0.005
Bradyrhizobium namibiense 5-10^T	3. Namibia, Mashare, MADI Res. Station S17.89274, E20.21068	7.6	96	0.42	8.6	35.4	0,174
Bradyrhizobium sp. 1-7	4. Namibia, Mashare, MADI Res. Station S17.895486, E20.211047	6.4	17	0.42	11.1	0.009	0.026
Bradyrhizobium sp. WR23, WR27	5. Angola, Bié Province, Miombo woodlands near Cusseque S13.69972, E17.06894	5.5	13	0.791	16.1	0.008	n.d.
Ensifer sp.WR41	6. South Africa, Namaqualand S30.385722, E 18.28516	5.8	22	0.31	10.3	n.d	n.d.
Mesorhizobium sp. WR52	6. South Africa, Namaqualand S30.385722, E 18.28516	5.8	22	0.31	10.3	n.d	n.d.
Bradyrhizobium sp. WR74	7. South Africa, Namaqualand S30.385722, E 18.28517	n.d.	n.d.	n.d.	n.d.	n.d	n.d.

Bradyrhizobium sp. WR93	2. Namibia, Nkwazi Lodge S17.515515, E19.542997	5.4	36	1.37	11.6	0.057	0.005
Microbacterium sp. WR95	8. Namibia, Nursery in Rundu	n.d.	n.d.	n.d.	n.d.	n.d.	n.d.
Bradyrhizobium sp. WR96	9. Namibia, Kalahari near street Gobabis-Drimiopsis, S22.290000, E018.99632	n.d.	n.d.	n.d.	n.d.	n.d.	n.d.

[a] Soil data from SASSCAL Data and Information Portal, https://www.sasscal.org/sasscal-data-and-information-portal/

[b] Numbering as in Table S1, Location/coordinates

Supplementary Table S3 | PCR conditions and accession numbers for genes sequenced in this study[a]			
**Gene**	**Sequence with accession numbers**	**primer names with sequence**	**Conditions for amplification by PCR**
16S rRNA	*Bradyrhizobium ripae* strain WR4[T] (**MF593081**); *Bradyrhizobium* sp. WR23 (**MK259093**); *Bradyrhizobium* sp. WR74 (**MN174665**); *Bradyrhizobium* sp. WR93 (**MK259089**); *Bradyrhizobium* sp. WR96 (**MN174665**); *Bradyrhizobium vignae* 7-2 (**KP899563**); *Ensifer* sp. WR41 (**MK259091**); *Mesorhizobium* sp. WR52 (**MK259092**)	Forward primer Bac8uf (5'AGAGTTTGA TNHTGGYTCAG 3'); reverse primer Univ1492r (5'GGNTACCTT GTTACGACTT3')	95°C - 5 min; 40 cycles of 95°C - 1 min, 50°C - 30 s, 72°C - 2 min; final elongation 72°C - 10 min
ITS: 16S-23S rRNA internal transcribed spacer region	*Bradyrhizobium ripae* strain WR4[T] (**MF593082**); *Bradyrhizobium* sp. WR23 (**MH171245**); *Bradyrhizobium* sp. WR74 (**MH171175**); *Bradyrhizobium* sp. WR93 (**MH171247**); *Bradyrhizobium* sp. WR96 (**MH171260**); *B. vignae* 7-2 (**KM378574**); *B. kavangense* 14-3 (**KM378507**); *B. subterraneum* 58 2-1 (**KM378539**); *Bradyrhizobium* sp. 1-7 (**KM378498**); *B. namibiense* 5-10 (**KM378502**)	Forward primer FGPS132' (5'CCGGGTTTCC CCATTCGG- 3'); reverse primer FGPS1490 (5'TGCGGCTGG ATCACCTCCTT 3')	95°C - 4 min; 40 cycles of 94°C - 1 min, 58°C - 1 min, 72°C - 2 min; final elongation 72°C - 10 min
glnII	*Bradyrhizobium ripae* strain WR4[T] (**MF593086**); *Bradyrhizobium* sp. WR23 (**MK689367**); *Bradyrhizobium* sp. WR74 **(MH182945**); *Bradyrhizobium* sp. WR93 (**MH182984**); *Bradyrhizobium* sp. WR96 (**MH182991**); *B. vignae* 7-2 (**KM378443**); *B. kavangense* 14-3 (**KM378446**); *B. subterraneum* 58 2-1 (**KM378484**); *Bradyrhizobium* sp. 1-7 (**KM378436**); *B. namibiense* 5-10 (**KM378440**)	Forward primer glnII12F (5'YAAGCTCGA GTACATYTGGC T3'); reverse primer glnII689R (5'TGCATGCCSG AGCCGTTCCA3')	95°C - 5 min; 35 cycles of 94°C - 1 min, 57°C - 40 s, 72°C - 1 min; final elongation 72°C - 10 min
recA	*Bradyrhizobium ripae* strain WR4[T] (**MK689368**); *Bradyrhizobium* sp. WR23	Forward primer recA41F	95°C - 5 min; 35 cycles of 94°C - 1

	(**MK689368**); *Bradyrhizobium* sp. WR74 (**MH182760**); *Bradyrhizobium* sp. WR93 (**MH182814**); *Bradyrhizobium* sp. WR96 (**MH182797**); *B. vignae* 7-2 (**KM378374**); *B. kavangense* 14-3 (**KM378399**); *B. subterraneum* 58 2-1 (**KM378397**); *Bradyrhizobium* sp. 1-7 (**KM378372**); *B. namibiense* 5-10 (**KM378377**)	(5’TTCGGCAAGGGMTCGRTSATG3’); reverse primer recA640R (5’ACATSACRCCGATCTTCATGC3’)	min, 57°C - 40 s, 72°C - 1 min; final elongation 72°C - 10 min
gyrB	*Bradyrhizobium ripae* strain WR4^{T} (**MF593094**); *Bradyrhizobium* sp. WR23 (**MH182827**); *Bradyrhizobium* sp. WR74 **(MH182865**); *Bradyrhizobium* sp. WR93 (**MH182838**); *Bradyrhizobium* sp. WR96 (**MH182815**); *B. vignae* 7-2 (**KX683216**); *B. kavangense* 14-3 (**KX661397**); *B. subterraneum* 58 2-1 (**KX661396**); *Bradyrhizobium* sp. 1-7 (**MK689365**); *B. namibiense* 5-10 (**KX661393)**	Forward primer gyrB343F (5’GAAYTCCTAYAAGG3’); reverse primer gyrB1043R (5’AGCTTGTCCTTSGTCTGCG3’)	95°C - 5 min; 5 cycles of 94°C - 2 min, 57°C - 2 min, 72°C - 1.5 min; then 28 cycles of 94°C - 30 s, 57°C - 1 min, 72°C - 1.5 min; final elongation 72°C - 5 min
nodC	*Bradyrhizobium ripae* strain WR4^{T} (**MF593106**); *Bradyrhizobium* sp. WR23 (**MK259093**); *Bradyrhizobium* sp. WR74 (**MK259095**); *Bradyrhizobium* sp. WR93 (**MK259094**); *Bradyrhizobium* sp. WR96 (**MK259096**); *B. vignae* 7-2 (**KT362339**); *Bradyrhizobium* sp. 1-7 (**MK259097**); *B. namibiense* 5-10 (**KX661399**); *B. diazoefficiens* USDA 110 (**BA000040**); *B. yuanmingense* NBRC100594 (**AB354633**); *B. tropiciagri* SEMIA 6148 (**KP234520**); B. lablabi CCBAU23086 (**GU433565**); *B. elkanii* USDA 76 (**HQ233221**); *Ensifer* sp. WR41 (**MW353154**); *Mesorhizobium* sp. WR52 (**MW353155**)	Forward primer NodCfor540 (5'TGATYGAYATGGARTAYTGGCT3'); reverse primer NodCrev1160 (5'CGYGACARCCARTCGCTRTTG3')	95°C - 4 min; 35 cycles of 94°C - 1 min, 51°C - 1 min, 72°C - 1 min; final elongation 72°C - 10 min

[a] List of GenBank accession numbers (bold) for the marker gene sequences obtained for this study. Primer names and sequences as well as PCR amplification conditions given for each marker gene.

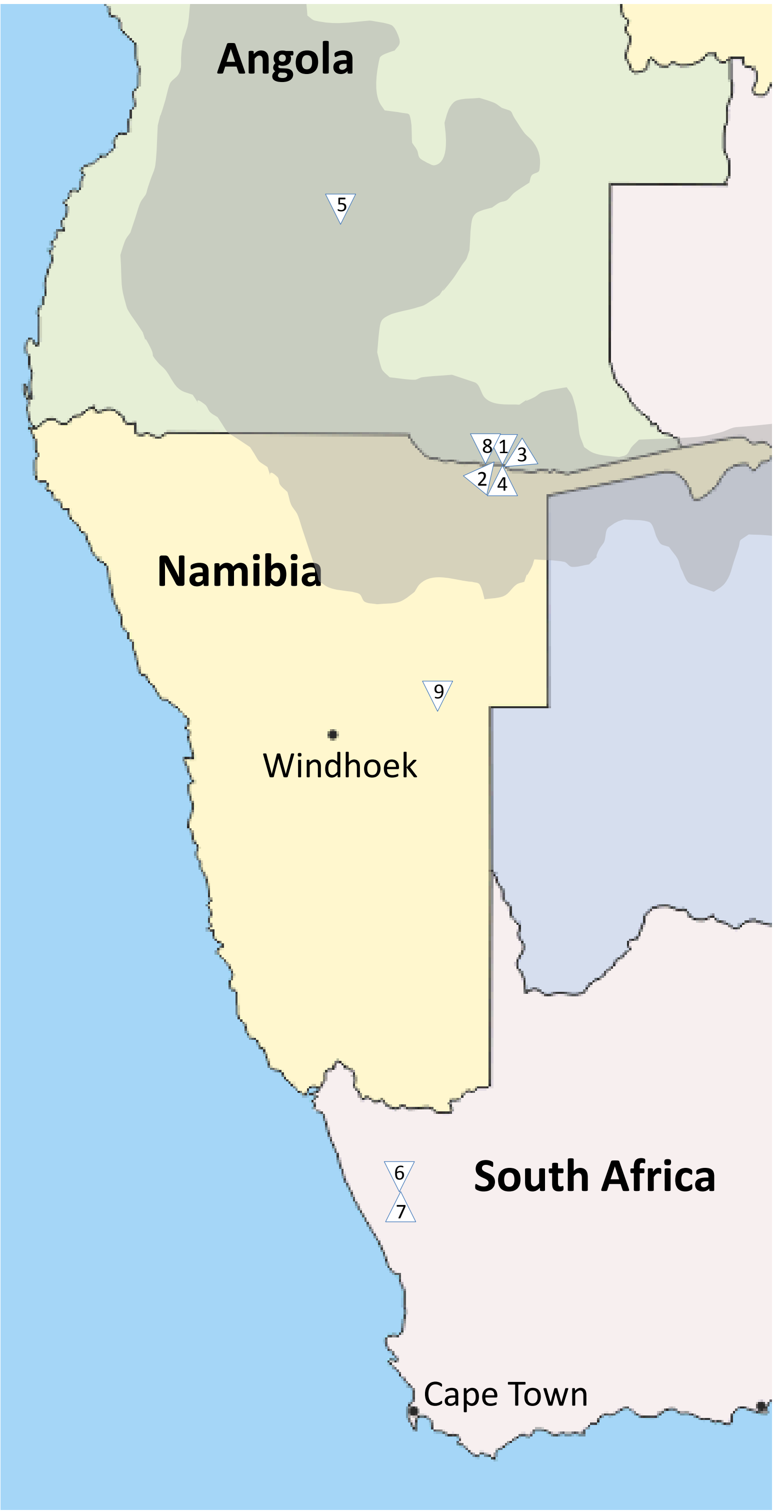

Supplementary Figure S1 | Map of sampling locations. Triangles point to sampling locations numbered according to Table S1. Gray shade, distribution of *Pterocarpus angolensis* according to De Cauwer et al. (2017) Map from d-maps.com.

De Cauwer, V., Fichtler, E., Beeckman, H., Graz, F.P., Mertens, J., Van Holsbeeck, S., et al. (2017). Predicting site productivity of the timber tree *Pterocarpus angolensis*. *South. Forests* 79**,** 259-268. doi: 10.2989/20702620.2016.1256042.

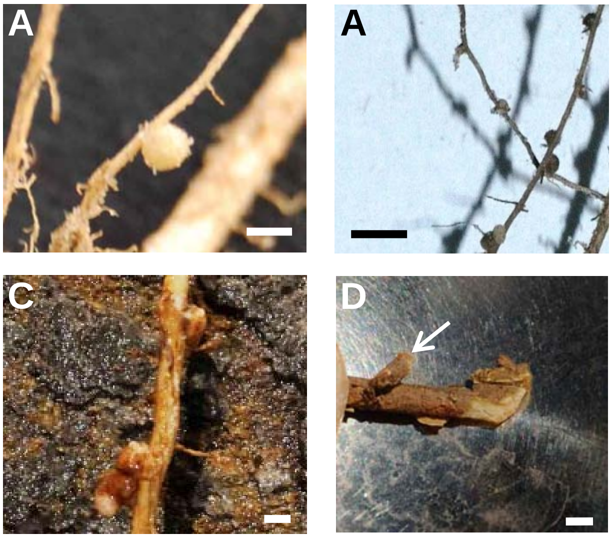

Supplementary Figure S2 | Root nodules found in plants collected for isolation of root nodule bacteria. **(A)** *Desmodium barbatum*, **(B)** *Indigofera rautanenii*, **(C)** *Chamaecrista* sp., **(D)** *Wiborgia monoptera.* Bars represent 2 mm.

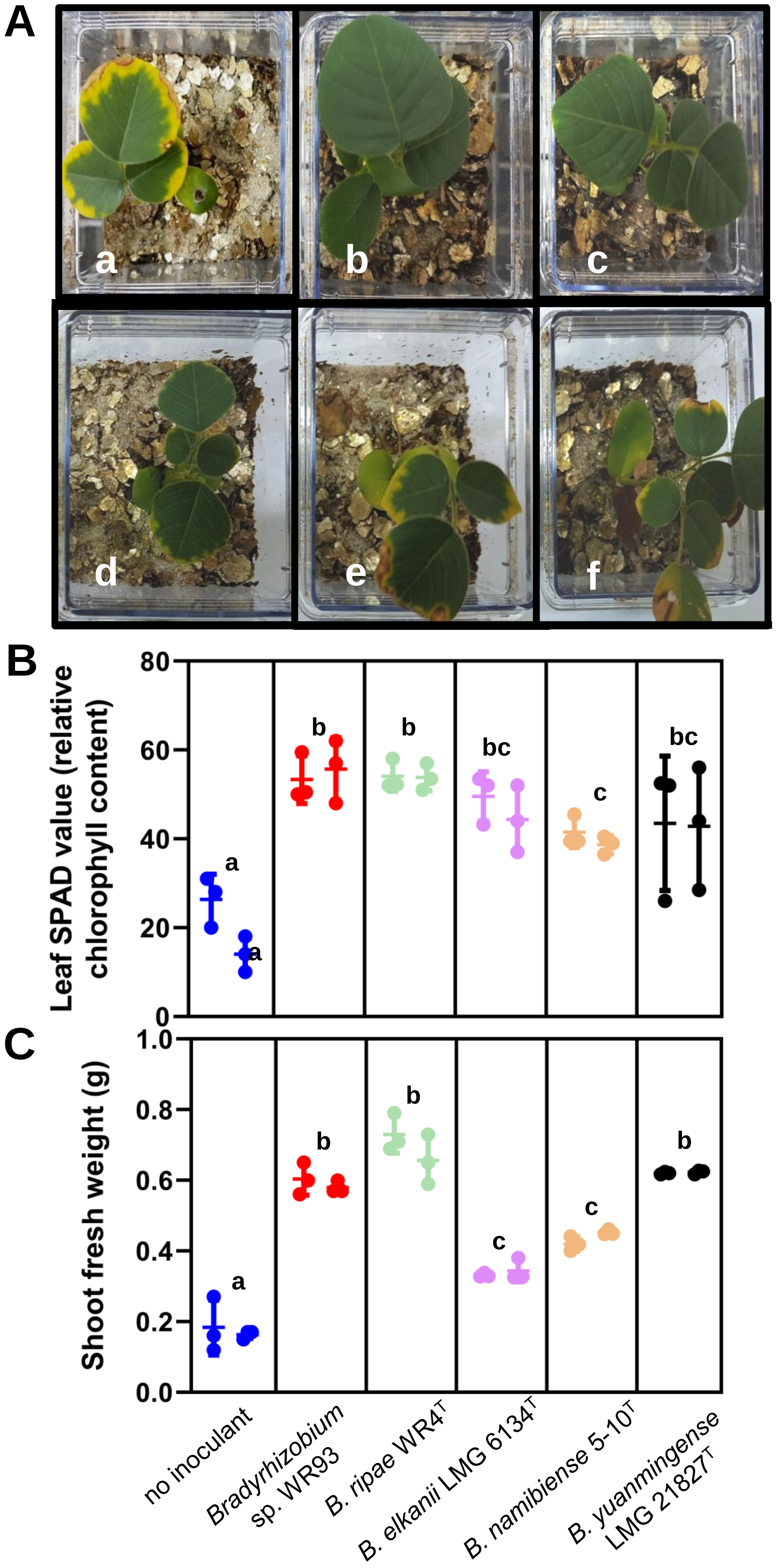

Supplementary Figure S3│*Pterocarpus angolensis* phenotypes (A) leaf SPAD values (B) and shoot fresh weight (C) 6 weeks post inoculation; growth in sterile vermiculite/sand mixture in

phytotron. (A) *Pterocarpus angolensis* with (a) no inoculant (negative control) or treated with following different inoculants: *Bradyrhizobium* sp. WR93 (b) , *B. ripae* WR4^{T} (c), *B. elkanii* LMG 6134^{T} (d), *B. namibiense* 5-10^{T} (e), or *B. yuanmingense* LMG 21827^{T} (f). (B) Soil-plant analysis development (SPAD) meter values of young leaves from inoculated or non-inoculated plants before harvest Three SPAD values each from three young leaves were recorded per plant. Data from two independent inoculation experiments with three plants each, shown separately. © hoot fresh weights of the same experiment. Scatter dot plots shown with means (horizontal line), standard deviation (bars), and original values as dots. Data with different letters indicate statistic significance ($P < 0.05$) between treatments, using an ANOVA mixed-effects model (GraphPad Prism 9.0).

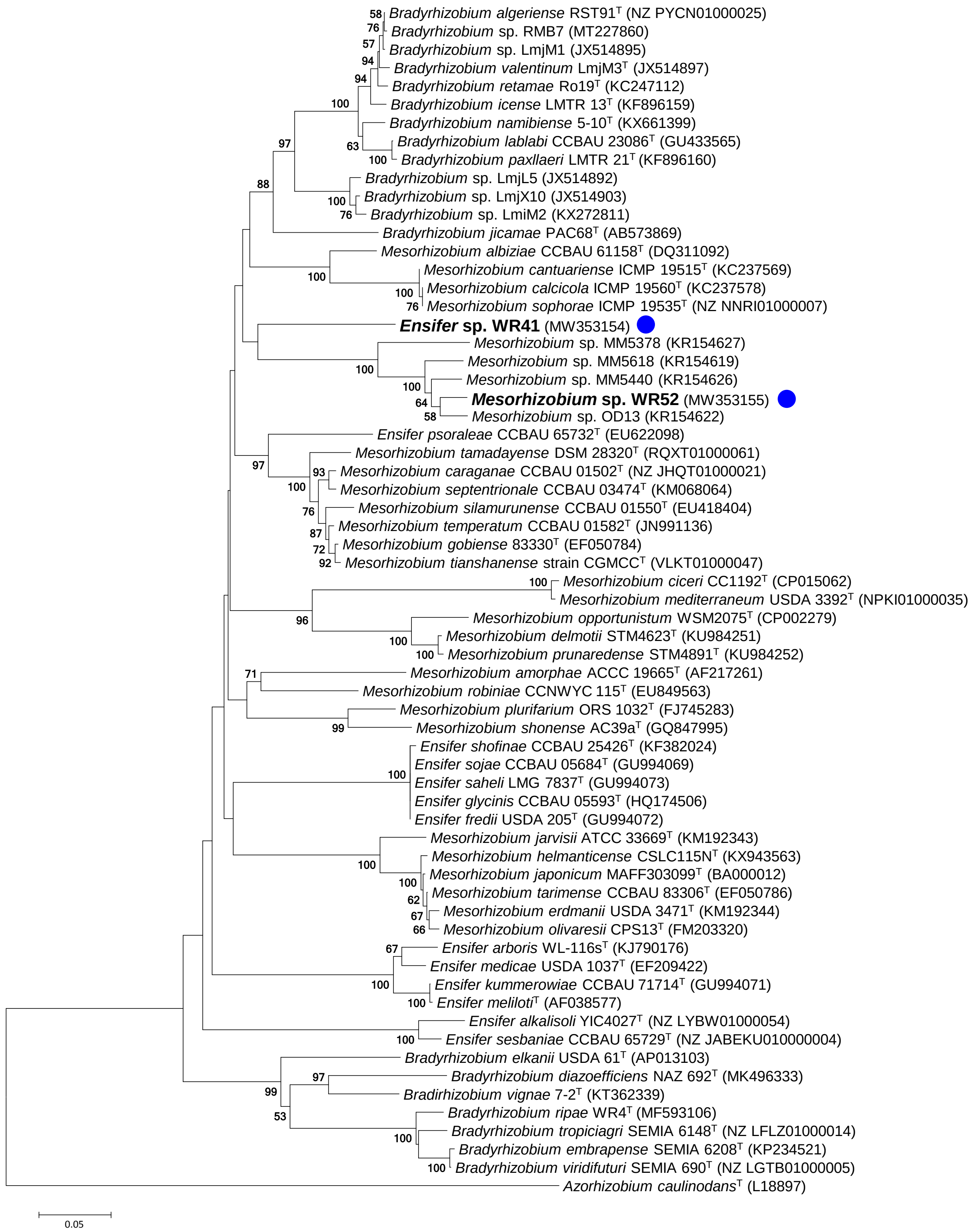

Supplementary Figure S4 | Neighbor-joining phylogram of partial *nodC* nucleotide sequences *Mesorhizobium* sp. WR52 and *Ensifer* sp. WR41 isolated from root nodules of *Wiborgia monoptera.*

The evolutionary history was inferred using the Neighbor-joining method. The percentage of replicate trees in which the associated taxa clustered together in the bootstrap test (1000 replicates) are shown next to the branches. The evolutionary distances were computed using the Maximum Composite Likelihood method and are in the units of the number of base substitutions per site. The analyses involved 65 nucleotide sequences of isolates (in bold and marked by blue dots) and reference strains with a total of 521 positions in the final dataset. Evolutionary analyses were conducted in MEGA 6.
